# Risk factors for cognitive decline in persons with HIV

**DOI:** 10.1097/QCO.0000000000001080

**Published:** 2024-12-06

**Authors:** Merle Henderson, Alan Winston

**Affiliations:** a Department of Infectious Disease, Faculty of Medicine, Imperial College London; b Jefferiss Wing, St Mary's Hospital, Imperial College Healthcare NHS Trust, London, UK

**Keywords:** aging, antiretroviral therapy, cognition, comorbidities, HIV, risk factors

## Abstract

**Purpose of review:**

Cognitive disorders persist in persons with HIV, despite virologically suppressive antiretroviral therapy. We summarize the current evidence on risk factors for cognitive decline in persons with HIV in the modern antiretroviral therapy-era.

**Recent findings:**

Recent consensus recommendations have proposed a new approach for defining cognitive impairment in persons with HIV, which distinguishes true cognitive impairment from low cognitive performance alone and considers both HIV and non-HIV-associated causes of brain injury. Adverse mental health, risks associated with substance misuse, and an increasing burden of age-related comorbidities have been highlighted as important contributors toward cognitive decline in this population. Aging may potentiate these risk factors through polypharmacy and drug-drug interactions.

**Summary:**

Cognitive decline in persons with HIV is likely multifactorial, with contributions from both HIV and non-HIV-associated mechanisms, particularly age-related comorbidities. With an aging community of persons with HIV, screening for risk factors associated with cognitive decline may be crucial to implement appropriate risk reduction strategies.

## INTRODUCTION

Cognitive disorders secondary to HIV-disease have been well recognized since the start of the HIV-epidemic. Prior to the introduction of antiretroviral therapy (ART), HIV-dementia was observed in over 50% of persons with advanced HIV [1]. With modern ART, which can suppress the virus to undetectable levels in both the plasma and cerebrospinal fluid, severe HIV-associated brain disease is now rare. However, despite virologically suppressive ART, milder forms of cognitive impairment persist in persons with HIV [2]. Such impairments may significantly impact on quality of life for those affected and may pose specific challenges, including an individual's ability to adhere to medication [3].

The underlying mechanisms driving mild cognitive disorders in well treated people with HIV are likely multifactorial. Suggested mechanisms include the direct effects of HIV on the brain, including persistent immune activation and neuroinflammation despite virologically suppressive ART, the legacy effects of central nervous system damage (CNS) from uncontrolled HIV-replication sustained prior to the initiation of ART, and ART toxicity [4,5]. With the availability of effective, virologically suppressive ART, other non-HIV associated mechanisms are increasingly recognized as causes of cognitive impairment and include the effects of comorbid conditions, such as vascular disease and mental health conditions [6].

As the community with HIV age, the prevalence of cognitive impairment and other age-related comorbidities will continue to increase. To implement appropriate risk reduction measures, it is vital we understand the *current* mechanisms toward the development of cognitive impairment in persons with HIV. In this review we summarize the up-to-date evidence on risk factors for cognitive decline in persons with HIV in the modern ART-era. 

**Box 1:**
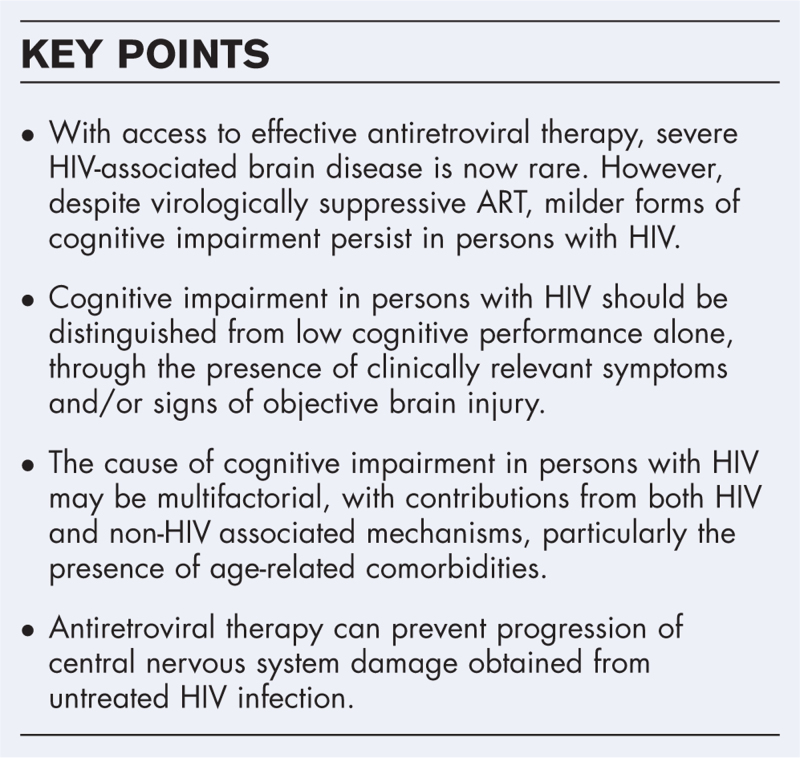
no caption available

## CLASSIFICATION OF BRAIN HEALTH AND BURDEN OF COGNITIVE DISORDERS IN PERSONS WITH HIV

Historically, the HIV-associated neurocognitive disorders (HAND) criteria, also known as the Frascati criteria, have been used for the classification of cognitive disorders in persons with HIV. On the basis of low performance on cognitive testing [≥1.0 standard deviations (SD) below the normative mean in two or more cognitive domains], individuals are categorized into groups based on the presence or absence of symptoms: asymptomatic vs. symptomatic neurocognitive impairment (NCI). Symptomatic NCI is further subcategorized into mild or severe NCI (HIV-associated dementia; ≥2.0 SD below the normative mean in two or more cognitive domains) [7]. These criteria have several limitations: Firstly, they do not account for complex cultural and psychosocial factors, which may impact on cognitive performance, in the absence of disease; Secondly, a high false-positive rate may over-diagnose cognitive impairment in persons with HIV, leading to undue anxiety and stigma [8,9^▪▪^]. Using these criteria, up to 60% of persons with HIV may be classified as impaired; findings not in keeping with rates observed in clinical practice [9^▪▪^]. In recognition of these limitations, new consensus recommendations were proposed by the International HIV-Cognition Working Group (2023) for the diagnosis and classification of cognitive impairment in persons with HIV [9^▪▪^]. Broadly, this approach recommends that a classification of cognitive impairment should be distinguished from low cognitive performance alone through the presence of clinically relevant symptoms and/or signs of objective brain injury. Importantly, these recommendations highlight the need to consider *all* causes of brain injury in persons with HIV, to distinguish HIV and non-HIV-related causes of cognitive decline [9^▪▪^].

Given there has been no consensus in definition of cognitive impairment in persons with HIV, estimating the disease prevalence is challenging. As noted above, if using the HAND criteria, high prevalence rates are observed. Using only clinical criteria, prevalence rates of cognitive disorders are reported to be between 2 and 5% [10]. As cognitive function assessments are variable, describing incident cognitive impairment is challenging and in some individuals cognitive function improves, while in others it deteriorates [6].

## HIV-ASSOCIATED BRAIN DISEASE

HIV has been detected in the cerebrospinal fluid as early as 8 days after HIV exposure [11], which may be secondary to trafficking of infected CD4^+^ T cells and monocytes across the blood–brain barrier, as well as potential free virus entry [12]. HIV infection and activation of resident CNS immune cells may cause neuroinflammation and injury through the release of viral proteins, neurotoxins, reactive oxygen species, and inflammatory proteins [12,13].

Legacy HIV-associated brain injury (HABI) refers to CNS damage caused by the direct effects of untreated HIV infection occurring prior to commencing effective ART [9^▪▪^]. Once established, this damage may be irreversible, and ART may not ameliorate these effects; data from a U.S. cohort of men with HIV with cognitive impairment demonstrated that cognitive function did not improve following ART introduction over a 15-year follow-up period [5]. Reassuringly, with ART, these legacy effects on cognition were static, and cognitive performance did not worsen during the study period. In line with consensus recommendations from the International HIV-Cognition Working Group, legacy HABI should be distinguished from active HABI; ongoing and progressive brain injury (clinical or radiological), which may occur in the presence or absence of HIV RNA suppression [9^▪▪^]. Active HABI in the presence of plasma HIV RNA suppression may be secondary to cerebrospinal fluid HIV RNA escape, where there is detectable HIV RNA in the cerebrospinal fluid at concentrations greater than in the plasma. Cerebrospinal fluid HIV RNA escape may present with neurological symptoms including cognitive decline, dizziness, and headaches [14]. The presence of cerebrospinal fluid HIV RNA escape has recently been observed in up to 17% of persons with HIV undergoing cerebrospinal fluid examination for clinical indications between 2017 and 2022 [14]. This frequency was unchanged in recent years when compared to historical data, despite the availability of modern ART, highlighting the need for an ongoing vigilance for cerebrospinal fluid HIV RNA escape in neurologically symptomatic persons with HIV [14]. While the overall prevalence of cerebrospinal fluid HIV RNA escape is rare in persons with HIV without symptoms, it can also be asymptomatic and transient, equivalent to a plasma viral load blip, or secondary to other CNS infections. Symptomatic cerebrospinal fluid escape requires prompt management to optimize ART in the context of cerebrospinal fluid/plasma genotypic resistance profiles [15].

Data remain limited on whether active HABI can occur in the presence of plasma and cerebrospinal fluid HIV RNA suppression, which has been hypothesized as secondary to ongoing low-grade CNS viral replication or neuroinflammation. In a recent placebo-controlled, randomized controlled trial, ART intensification with dolutegravir or dolutegravir and maraviroc did not improve cognitive performance in a cohort of persons with suppressed HIV with cognitive impairment over a 96-week period. While these findings do not support the hypothesis of ongoing low-grade CNS viral replication, these results should be interpreted with caution. Individuals with Frascati-defined *asymptomatic* NCI comprised 35% of the study population, which in some individuals may represent low cognitive performance alone rather than true cognitive impairment, raising concerns that this was not the appropriate population to study the potential benefit of this intervention. Similarly, the study criteria did not distinguish between legacy and active HABI, or cognitive impairment due to other non-HIV associated causes. Taken in combination, this study highlights the importance of using contemporary, uniform definitions for defining cognitive impairment across research settings. Whether active HABI can occur in the presence of plasma and cerebrospinal fluid HIV RNA suppression remains an important consideration for further research efforts [16].

### Antiretroviral therapy-associated neurotoxicity

Optimal penetration of ART in the CNS is required to maintain the balance of viral control vs. the prevention ART-related neurotoxicity, both of which may contribute to cognitive decline. Cerebrospinal fluid is typically used as a surrogate marker for CNS drug exposure, due to the invasive nature of brain tissue biopsy. However, evidence suggests cerebrospinal fluid drug concentrations may not be reflective of brain tissue concentrations. A recent postmortem study from Uganda measured ART concentrations (dolutegravir, tenofovir, lamivudine, and efavirenz) collected from plasma, cerebrospinal fluid, and brain tissue in a cohort of 49 participants [17]. Regional CNS differences in ART distribution were identified, which varied according to the individual drug. Overall, brain tissue concentrations were lower than cerebrospinal fluid concentrations for dolutegravir, tenofovir, and lamivudine. While tenofovir and lamivudine generally had brain concentrations that fell within the in-vitro EC_50_ range, both dolutegravir and efavirenz had brain tissue concentrations that were above the highest EC_50_ (dolutegravir) or EC_90-95_ (efavirenz), which may be a contributing factor toward a higher risk of neuropsychiatri side effects with these agents. Notably, participants included in this study were critically ill, with significant disease, which may have impacted on blood--brain barrier integrity and CNS penetration of these drugs. Further studies in a more generalizable population are required.

ART-associated neuropsychiatric side effects are well recognized and occur with several of the current ART drug classes. The nonnucleoside reverse transcriptase inhibitor (NNRTI) efavirenz has been linked to a considerable CNS adverse side effect profile [18], with an improvement in CNS symptoms and cognitive function demonstrated in persons with HIV switching from efavirenz to other nonefavirenz based ART regimens [19–22]. Similarly, neuropsychiatric side effects related to the use of integrase strand transfer inhibitors (INSTIs) have also been reported in persons with HIV, the frequency of which may vary according to the specific agent used; a recent U.K. study demonstrated improvements in subjective (patient-reported symptoms) and objective (functional connectivity within resting state cerebral functional MRI networks) measures of cerebral function in persons with HIV with neuropsychiatric side effects (insomnia) switching from a dolutegravir to a bictegravir-containing ART regimen over a 120-day follow-up period [23]. The causative mechanisms behind INSTI-related neuropsychiatric side effects are not well understood. Data suggest toxicities are not dose-dependent toxicity [24] and may be potentiated by aging, possible through the effects of polypharmacy and an increased risk of drug-drug interactions. Other mechanisms, including the interaction between specific genetic variants and preexisting psychiatric conditions, warrant further investigation [25].

## NON-HIV-ASSOCIATED BRAIN DISEASE

The cause of cognitive impairment in the modern ART-era is often multifactorial, and the effects of HIV on the brain may coexist alongside other comorbidities, which themselves may cause or contribute to cognitive decline in persons with HIV.

### Mental health

Adverse health-related quality of life, with significantly higher rates of depression and anxiety, are reported in persons with HIV when compared to persons without HIV [26]. Depression may be an independent risk factor for cognitive impairment, as well as a confounding factor in its assessment. Several pathogenic mechanisms underlying the increased risk of mental health conditions have been suggested, including the potential role of neuroinflammation, which has been liked to depressive illnesses in persons without HIV [27]. A recent study by Mudra Rakshasa-Loots *et al*. [28] observed in a cohort of virologically suppressed persons with HIV (*n* = 125), HIV was associated with both a higher prevalence of depressive symptoms (26 vs. 11%, *P* = 0.02), and elevated concentrations of systemic (plasma MIG and TNF-α) and central (cerebrospinal fluid MIP1-α and IL-6) biomarkers of inflammation, when compared to a lifestyle-similar group of persons without HIV (*n* = 79); findings which support the hypothesis that depressive symptoms may be in part mediated by ongoing inflammation in persons with HIV.

18F-fluorodeoxyglucose (FDG)-PET is a form of noninvasive imaging, which can detect vascular inflammation through uptake of radiotracers (18F-FDG) within inflamed arterial walls [29]. A cross-sectional study by Chow *et al*. [30] examined the relationship between psychological stress, depression, and arterial inflammation in 37 virologically suppressed persons with HIV (mean age 60 years, 97% men) through patient-reported outcome measures (PROMs), 18F-FDG-PET, and plasma biomarkers of immune activation and inflammation. Among persons with HIV, PROMs of stress correlated with biomarkers of systemic inflammation (perceived stress and hsCRP *r* = 0.33, chronic stress with IL-6 and sCD163, *r* = 0.33 and 0.35, respectively; all *P* < 0.05). Perceived stress was associated with carotid inflammation (*r* = 0.41, *P* = 0.023) independent of age, race/ethnicity, traditional vascular risk factors, and health-related behaviors such as substance misuse [30]. Carotid inflammation, measured by 18F-FDG-PET, has been independently associated with lower global cognitive function in virologically suppressed persons with HIV; findings which persisted after adjustment for confounding factors, including cardiovascular disease risk (*r* = -0.34, *P* = 0.029) and measures of posttraumatic stress (*r* = -0.33, *P* = 0.033) [31]. However, these findings were not reflected in those with aortic inflammation and further longitudinal studies are needed to evaluate the relationship between vascular inflammation and the development of cognitive impairment over time.

### Central nervous system aging and age-related comorbidities

Aging is a recognized risk factor for cognitive decline across all populations. Accelerated epigenetic aging has also been demonstrated in persons HIV, associated with both HIV-disease severity (lower current and nadir CD4^+^ cell counts, and previous AIDS diagnoses) and psychosocial factors (health literacy), which may impact on healthcare engagement [32,33]. Age-related chronic comorbidities, such as cardiovascular disease, are also more common in persons with HIV and can occur at an earlier age, when compared to those without HIV [34]. Such comorbidities may further accelerate biological and CNS aging, in-turn increasing the risk of cognitive decline.

### Cerebrovascular disease and stroke

Women with HIV may be disproportionally affected by cerebrovascular disease and stroke, both known risk factors for the development of cognitive impairment. In a recent observational cohort study of cerebrovascular risk in more than 13 000 persons with HIV living in the U.S., individuals of female sex aged 50 years or younger had a higher risk of stroke, when compared to men of the same age, which was associated with both traditional (treated hypertension) and nontraditional risk factors for cardiovascular disease (HIV viraemia and methamphetamine use); findings which persisted after adjustment for race and ethnicity [35^▪^]. While there was no group without HIV to compare incident stroke rates by age group and sex, and a lack of data on relevant sex and gender specific factors (menopause, hormone replacement therapy, and sex-affirming medications), these data highlight the potential contribution of traditional risk factors on stroke risk in younger women [35^▪^].

### Multimorbidity, frailty, and polypharmacy

An increasing burden of multimorbidity, defined as two or more health conditions, is being recognized in persons with HIV, which has been associated with both HIV factors (e.g. duration since HIV diagnosis) and other non-HIV-related characteristics (e.g. age, BMI) [36].

Multimorbidity is a risk factor for cognitive impairment, with follow-up data from the CNS HIV Antiretroviral Therapy Effects Research (CHARTER) study, demonstrating a decline in cognitive performance testing [Global Deficit Score (GDS) -0.28] in persons with HIV over a 12-year period, associated with comorbidity burden [6]. Of note, and in contrast to existing literature [37], increasing age in the CHARTER cohort was not related to cognitive decline. The lack of an association with age may, in part, be a result of legacy HABI, with the majority of individuals reporting a history of previous AIDS (74%), which may have led to accelerated biological aging in younger individuals, inconsistent with chronological aging [6]. In another longitudinal U.S. cohort of over 1000 persons with HIV, higher comorbidity burden was associated with faster rates of cognitive decline [38].

Longitudinal studies describing the trajectories of cognitive function report differing results. In the Comorbidity in Relation to HIV and AIDS (COBRA) cohort, no differences in trajectories of cognitive health were observed over a 2-year period between persons with HIV and without HIV [39]. Importantly, individuals without HIV in this study had similar lifestyle factors to those with HIV. On the converse, other studies, which have reported an accelerated trajectory of brain aging in persons with HIV, have generally included control populations without HIV who have lower comorbidity burdens, which may partly explain the accelerated signal observed in individuals with HIV [40].

With an increase in multimorbidity in persons with HIV, the use of multiple medications is common. Polypharmacy, defined as the use of five or more medications, increases the risk of drug-drug interactions and has been associated with significant adverse outcomes including impaired cognition, increased rates of frailty, recurrent falls, and fractures in those over 50 years of age [41–44]. Certain medications are associated with a greater risk of harm, including anticholinergic agents and those with sedative effects [45]. In a Swiss cohort study of more than 1000 older adults with mostly suppressed HIV [median age 70 (IQR 67–74), 82% male], polypharmacy was identified in 50%, with 20% on at least one anticholinergic medication. Use of an anticholinergic medication was independently associated with self-reported cognitive symptoms (OR = 2.51; 95% CI = 1.31–4.80) [46^▪^]. These findings have also been reflected in younger cohorts with HIV [45]. Cooley *et al*. [47] examined the association between anticholinergic medications, cognitive function, and structural brain integrity, measured by MRI, in persons with (*n* = 209) and without HIV (*n* = 95). Across the cohort, anticholinergic medications were associated with reduced cerebral function parameters, including cognitive performance and structural brain integrity. People with HIV with a high burden of anticholinergic use had reduced learning and executive function when compared to those without HIV with a high anticholinergic burden [47]. Those that reduced their anticholinergic burden had improvements in cerebral function parameters over a 2-year period [47]. These findings highlight the need for polypharmacy stewardship, to promote proper medication use and limit adverse side effects, by preventing inappropriate or unnecessary prescribing and rationalizing medications where possible.

### Risks associated with substance use

Opioid misuse disorder is a risk factor for cognitive decline in persons with HIV, which is an increasing public health concern. Recent evidence suggests that opiate misuse may potentiate HIV-related pathogenic neuroinflammation, predisposing individuals to age-related neurodegenerative changes [48]. Sexual risk taking, as a result of substance misuse, may promote the transmission of sexually transmitted infections that may invade the CNS such as, syphilis and hepatitis C, both of which may impact of cognition. The role of cannabis in contributing to cognitive decline in persons with HIV is debated [49]. A recent review by Ayoub *et al*. [49] surmised that while cannabis use may have beneficial anti-inflammatory properties, increased dosage and frequency of use may have detrimental effects on cognition in persons with HIV.

## FUTURE CONSIDERATIONS

Novel HIV-therapeutics are being explored in early phase clinical trials. Broadly neutralizing antibodies (bNAbs), targeting different HIV epitopes in the envelope gene, are being evaluated as long-acting alternatives to daily oral ART. Their large molecular size is challenging for providing effective delivery across the blood--brain barrier (BBB) and limited human data are available on their CNS penetration. A recent study presented at the 25th International AIDS Conference (Munich, 2024) demonstrated that the bNAb VRC01 was detectable in the cerebrospinal fluid of people with acute and previously treated HIV 2–16 days after infusion [50]. However, concentrations of VRC01 in the cerebrospinal fluid were significantly lower when compared to plasma concentrations (3-4 logs lower) [50]. Of concern, distinct viral populations have been demonstrated in brain tissue, when compared to blood, which demonstrated reduced susceptibility to bNAbs in predictive models [51]. These findings highlight the importance of close CNS monitoring, to evaluate the impact of novel HIV therapeutics and planned analytic treatment interruptions (ATIs) on the CNS. Research toward novel strategies that facilitate penetration of bNAbs across the BBB, which are both well tolerated and effective in preventing CNS viral replication, are crucial to prevent the development adverse CNS side effects.

**FIGURE 1:**
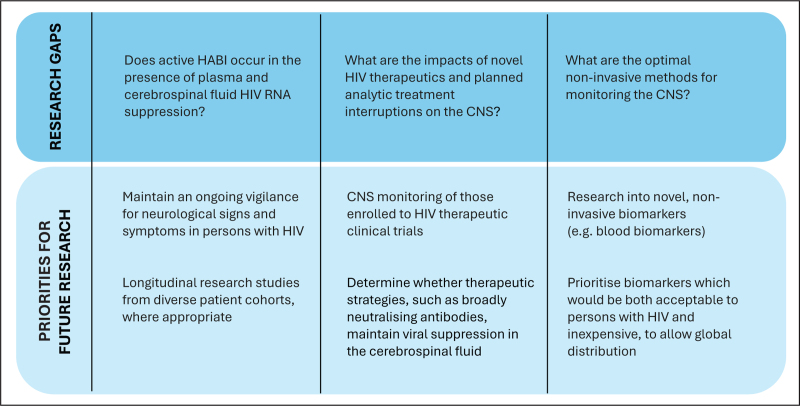
Research gaps and priorities.

Research gaps and priorities are summarized in Fig. 1.

## CONCLUSION

The available evidence highlights that cognitive disorders persist in persons with HIV in the modern ART era, despite virological suppression. Risk factors for cognitive decline include both HIV and increasingly, non-HIV associated mechanisms. With an aging community of persons with HIV, there is a growing burden of age-related comorbidities and multimorbidity, which may cause or contribute to cognitive decline. Aging may also potentiate ART toxicity through polypharmacy and an increased risk of drug-drug interactions. Research on cognitive impairment in historic cohorts of persons with HIV with legacy HIV-associated brain injury may not be truly reflective of the current profiles of cognitive impairment in persons with HIV. Harmonizing the approach toward cognitive impairment in persons with HIV, guided by new consensus recommendations, may help to provide more accurate prevalence estimates for cognitive impairment in the modern-ART era, reducing anxiety and stigma for persons with HIV. Crucially, screening for risk factors associated with cognitive decline in persons with HIV may help to identify individuals who may benefit from appropriate interventions.

## Acknowledgements

*None.*

### Financial support and sponsorship

*None.*

### Conflicts of interest

*Nil to disclose.*

## REFERENCES AND RECOMMENDED READING

Papers of particular interest, published within the annual period of review, have been highlighted as:

▪ of special interest
▪▪ of outstanding interest
